# Effects of Neonatal Neural Progenitor Cell Implantation on Adult Neuroanatomy and Cognition in the Ts65Dn Model of Down Syndrome

**DOI:** 10.1371/journal.pone.0036082

**Published:** 2012-04-25

**Authors:** Angela L. Rachubinski, Shannon K. Crowley, John R. Sladek, Kenneth N. Maclean, Kimberly B. Bjugstad

**Affiliations:** 1 Department of Pediatrics, School of Medicine, University of Colorado Denver, Anschutz Medical Campus, Aurora, Colorado, United States of America; 2 Departments of Exercise Science, and Neuropsychiatry and Behavioral Science, University of South Carolina, Columbia, South Carolina, United States of America; 3 Department of Neurology and Center for Neuroscience, School of Medicine, University of Colorado Denver, Anschutz Medical Campus, Aurora, Colorado, United States of America; 4 Colorado Intellectual and Developmental Disabilities Research Center (IDDRC), University of Colorado Denver, Anschutz Medical Campus, Aurora, Colorado, United States of America; Imperial College London, United Kingdom

## Abstract

As much of the aberrant neural development in Down syndrome (DS) occurs postnatally, an early opportunity exists to intervene and influence life-long cognitive development. Recent success using neural progenitor cells (NPC) in models of adult neurodegeneration indicate such therapy may be a viable option in diseases such as DS. Murine NPC (mNPC, C17.2 cell line) or saline were implanted bilaterally into the dorsal hippocampus of postnatal day 2 (PND 2) Ts65Dn pups to explore the feasibility of early postnatal treatment in this mouse model of DS. Disomic littermates provided karyotype controls for trisomic pups. Pups were monitored for developmental milestone achievement, and then underwent adult behavior testing at 14 weeks of age. We found that implanted mNPC survived into adulthood and migrated beyond the implant site in both karyotypes. The implantation of mNPC resulted in a significant increase in the density of dentate granule cells. However, mNPC implantation did not elicit cognitive changes in trisomic mice either neonatally or in adulthood. To the best of our knowledge, these results constitute the first assessment of mNPC as an early intervention on cognitive ability in a DS model.

## Introduction

The British physician John Langdon Down first described Down syndrome (DS) almost 150 years ago [1]. Today, DS is the most common genetic cause of intellectual disability and occurs in 1 in every 766 live births [2]. While other medical conditions associated with DS are treatable, intellectual disability remains the most limiting factor. Currently, no treatment can influence the proper cognitive development in DS and therefore provide life-long cognitive improvements. The potential of a neonatal intervention is appealing in DS, because many of the neuroanatomical abnormalities associated with DS have yet to develop. Small alterations in early development could affect the lifelong trajectory of development in the DS brain.

The triplication of genes in DS manifests in postnatal developmental delays and adult intellectual disability. In adulthood, individuals with DS are compromised in organizing and consolidating information, and in creating spatial maps [3]–[5]. These and other cognitive tasks are governed directly and indirectly by the hippocampus, a structure disproportionately affected in DS [5]–[7]. Hippocampal deficiencies include granule cell hypocellularity and abnormal synaptogenesis [8], [9]. These changes are present in fetal development, but because the hippocampus is dependent on early experiences to form synapses, synaptogenesis is not mature until childhood and continues to a lesser extent throughout life [10]. Proper hippocampal development is critical for cognitive development and function, making the hippocampus a promising structure to evaluate the impact of early intervention.

Trisomic Ts65Dn mice contain a partial triplication genes that are the murine homologues of human chromosome 21, the chromosome triplicated in DS [11]. The overexpression of analogous genes in the trisomic Ts65Dn mouse causes similar neuroanatomical and cognitive changes as in individuals with DS, including hindered neurogenesis, hypocellularity of granule cells in the dentate gyrus (DG), delays in developmental milestone achievement, impaired spatial abilities, and deficiencies in association and recognition memory [12]–[19]. The similarities between Ts65Dn mice and human DS make this model ideal to investigate the potential for early interventions.

Currently, NPC transplantation has proven to be of therapeutic value in the treatment of several adult neurodegenerative disorders including Parkinson's disease and ischemia. In these models, NPC were found to migrate to sites of damage, replace lost neurons and glia, and produce growth factors [20]–[27]. In adult Ts65Dn mice, we have previously found that NPC implanted into the hippocampus survived at least for one month and reduced the age-associated extracellular tau accumulation in the hippocampus [28], [29]. The adult neurodegenerative studies suggest a possible therapeutic potential for neonatal implantation of NPC as a way to influence the long-term cognitive outcome of DS. To test the effects of early neonatal NPC treatment in DS, we implanted murine NPC (mNPC) or a sham saline control into the hippocampus of neonatal Ts65Dn mouse pups. We assessed NPC survival and cellular changes in the dentate gyrus 16 weeks after implant. Behavior assessments were performed in the weanling period (Developmental Milestones) and 14–16 weeks post-implantation.

## Results

### Implanted mNPC Survival and Differentiation

Implanted mNPC were identified by immunohistochemistry against green fluorescent protein (GFP). GFP+ cells were found in the hippocampus and in areas outside the implant area in three out of six disomic animals and five out of six trisomic animals. Of the animals with evidence of surviving mNPC, the total numbers of mNPC suggest that the disomic group had almost three times more surviving mNPC than the trisomic group (t(33) = 6.87, p<0.0001). By extrapolating from the average number of cells found per section to the total number of possible sections, an estimated 5.56% of implanted mNPC survived in disomic brains. However, the survival rate of implanted mNPC was only 1.89% in trisomic brains.

**Figure 1:**
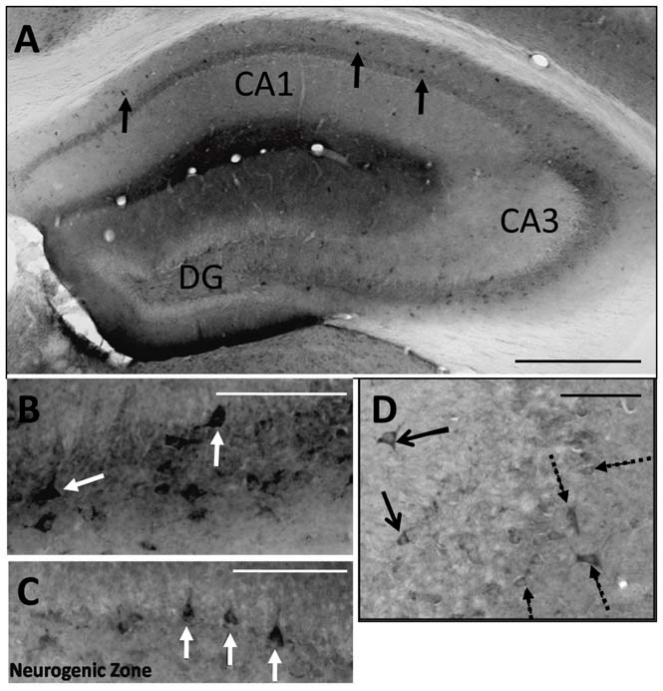
GFP+ cells found in the hippocampus of a 16 week disomic control brain. A. In this disomic brain, robust mNPC survival was observed with GFP+ cells found in throughout the hippocampus (black cells, arrows). B. mNPC found in the pyramidal layer of CA3 had the typical pyramidal neuronal shape (arrows). C. In the DG, mNPC were found in the subgranule zone, which is the neurogenic layer of the hippocampus (arrows). D. Double labeling for reelin revealed that some GFP+ cells found outside of the pyramidal layer of CA3 were reelin+ interneurons (solid arrows). Dashed arrows indicate host reelin+ interneurons. Scale bars in A = 500 µm and in B–D = 100 µm.

**Figure 2:**
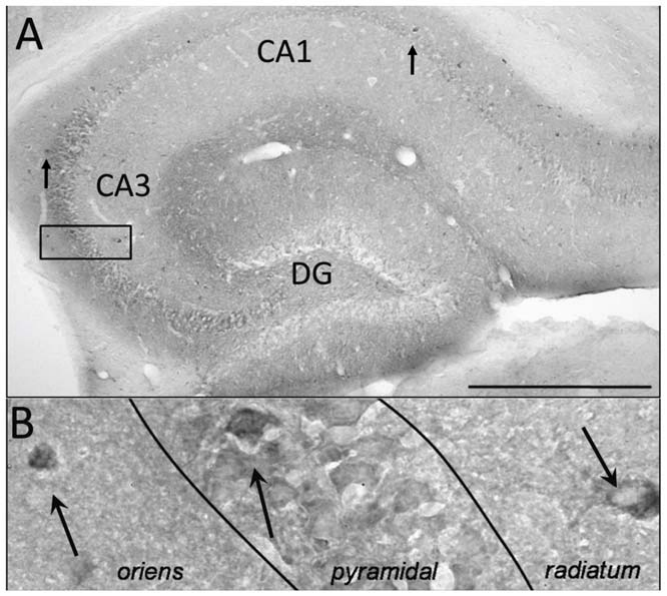
GFP+ cells found in the hippocampus of a 16 week trisomic brain. A. GFP+ cells (black cells, arrows) were most often found in CA1 and CA3. Scale bar = 500 µm. B. A magnification of the box in A, shows that GFP+ cells were found in the all three layers of CA3 (arrows).

In contrast, trisomic brains had better cell survival than disomic brains when only the GFP+ cells in the hippocampus were compared (t(18) = 7.23, p<0.0001). The hippocampus of trisomic brains contained 66.29% of all the identified implanted mNPC, whereas the disomic hippocampus only contained 42.01% of the identified cells. In the hippocampus, GFP+ cells were discernable in three of the disomic animals and four trisomic animals (Figures 1 and 2). The distribution of GFP+ mNPC in the hippocampus was similar between karyotypes, although the GFP+ cells were found in the pyramidal layers of disomic brains (Figure 1b) more often than in trisomic brains (Figure 2a,b). Some GFP+ interneurons were identified based on morphology and co-labeling with reelin in the stratum radiatum of disomic brains (Figure 1d) and in the oriens of trisomic brains. Attempts to co-label with neuronal marker, MAP2, or astrocyte marker, GFAP, found no GFP+ cells that double labeled for these markers of differentiation. Most GFP+ mNPC had the morphology of undifferentiated progenitor cells with small, compact soma without neuritic or glial processes.

**Figure 3:**
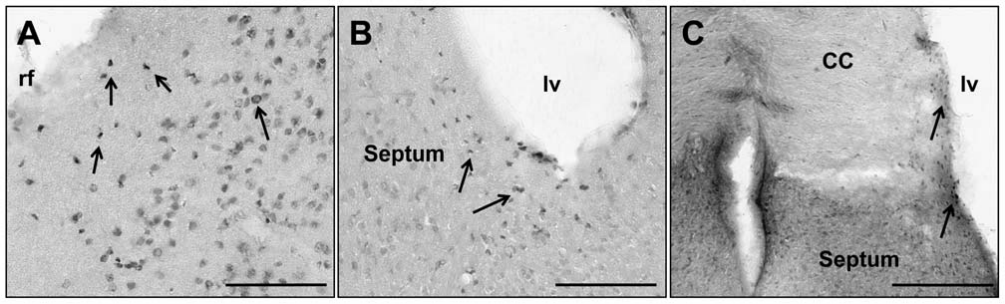
GFP+ cells found in areas outside of the hippocampus. A. Half of trisomic mice had GFP+ cells in the entorhinal cortex (arrows). B. GFP+ cells were found near the lateral ventricles in the septum (arrows). C. GFP+ mNPC also were found in the CC and again in the underlying septal areas (arrows). rf, rhinal fissure; lv, lateral ventricles; CC, corpus callosum. Scale Bars in A = 100 µm and in B–C = 200 µm.

The comparative differences in the number of implanted cells identified in the hippocampus, where the mNPC were implanted, and the rest of the brain indicates a disparity in the degree of migration from the implant site between the two karyotypes. The difference is in the number of mNPC that migrated and not the destination. In both karyotypes, GFP+ mNPC were identified in the lining of the lateral ventricles, including the neurogenic subventricular zone (SVZ) and endothelial lining adjacent to the septum, the corpus callosum, and the entorhinal cortex (Figure 3).

In summary, more trisomic brains were found to have surviving mNPC than disomic brains. However, of the brains with GFP+ cells, the number of surviving mNPC was greater in the disomic brain, where implanted mNPC migrated from the hippocampal site of implantation more often than in the trisomic brains.

### Granule Cell Density in the DG

**Figure 4:**
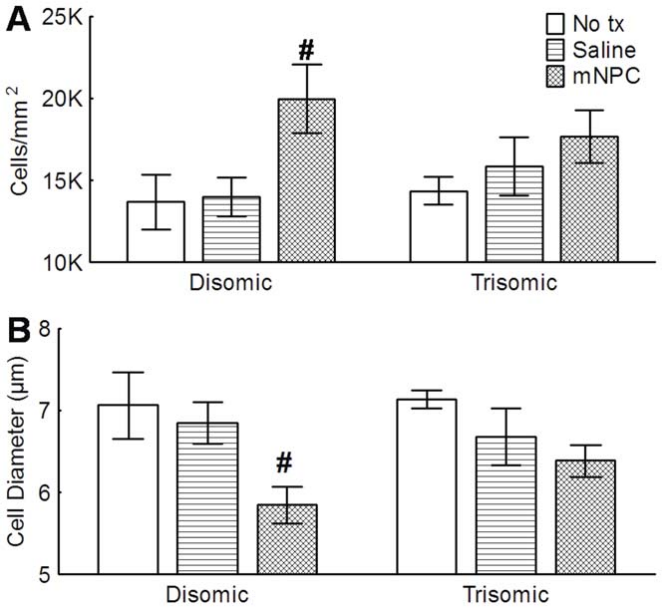
Treatment with mNPC significantly increased the density of the granule cells and decreased their diameter. A. There was no difference in the density of granule cells between disomic and trisomic mice overall. However, treatment with mNPC significantly increased the density of the granule cells (p<0.05). While this effect is more clearly observable in the disomic/NPC group (#), the interaction between treatment and karyotype suggests that mNPC effect was present in both karyotypes. B. Animals implanted with mNPC had granule cells with significantly smaller cell soma than untreated or saline treated animals (p<0.05). Again, this is best observed in the disomic group (#) although statistically both mNPC groups were affected. Mean ± SEM shown.

**Figure 5:**
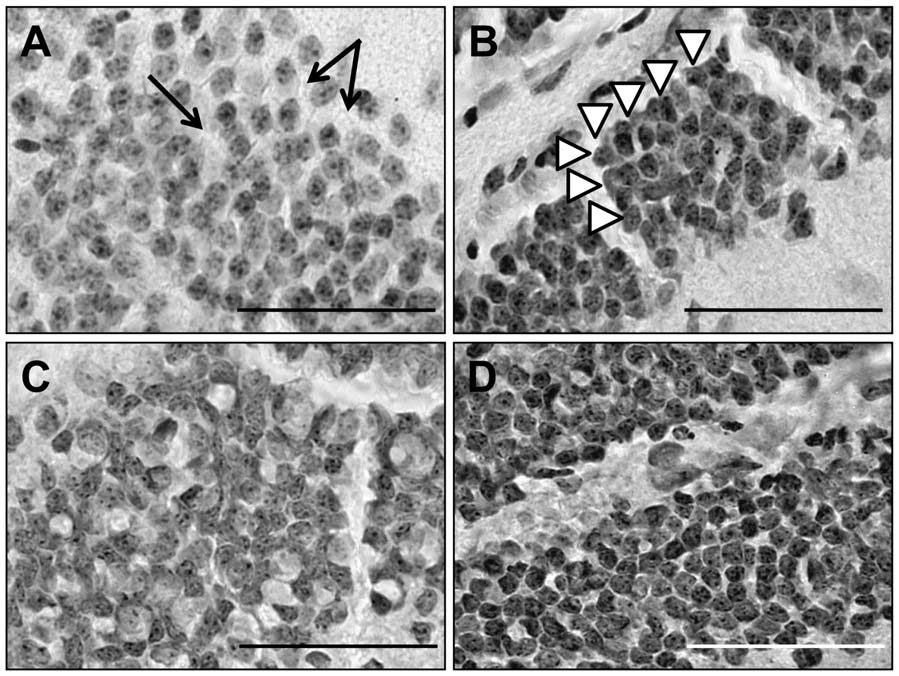
Organizational differences in the granule cells of the DG. Untreated disomic brains (A) had a lower density of granule cells, with a correspondingly larger cell diameter. Similar results were reported for untreated trisomic mice (C). In both groups, the granule cell layer appears less organized in that there was more space between the cells (arrows in A) and randomness to their location within the layer. In contrast, in the Disomic/mNPC mice (B) and Trisomic/mNPC mice (D), both of which had smaller cell diameters and a tendency towards a higher cell density, have a more organized appearance. The granule layers of these groups have tightly aligned cells, which sometimes appear to be in rows and columns (arrow heads in B). Scale bars in A–D are equal to each other and represent 100 µm.

While hypocellularity in the DG associated with DS has been previously reported, [16], [19], the current study did not find any differences in the granule cell density between the karyotypes (F(1, 32) = .003, p>0.05) (Figure 4a). However, the implantation of mNPC did significantly increase the density of granule cells by 33% compared to no treatment controls (F(2, 32) = 5.08, p<0.05). Treatment with saline did not have the same effect (p>0.05). Both karyotypes were affected equally by the implantation of mNPC (F(2, 32) = 0.86, p>0.05). The increased density of cells in the mNPC implanted brains may have resulted from the significant decrease in the diameter of the granule cells (F(2, 32) = 6.91, p<0.05). *Post hoc* analysis indicated that the average cell diameter was similar in untreated brains (7.1±0.19 µm) and saline implanted brains (6.8±0.2 µm). However, cell diameters in mNPC implanted brains were smaller than those in untreated brains by 13.8% (average diameter 6.1±0.19 µm) (Figure 4b). Again, the differences in density were not specific to one karyotype (F(2, 32) = 0.88, p>0.05). General observations of the DG depicts better organization in mNPC implanted groups as indicated by less space between cells and a column-like assembly (Figure 5).

### Neuroimmune Response to Implantation

Although the brain is a partially immunologically privileged site, it was important to ascertain if the host responses to the implantation procedure or mNPC differed between the karyotypes. Staining for microglia (Iba1) or astrocytes (GFAP) did not reveal a highly dense pattern of cells associated with an implant track in either saline or mNPC implanted animals of either karyotype (Figures S1 and S2). The lack of glial scarring and the similarity in resident populations of microglia and astrocytes between implanted and unimplanted groups indicated no long-term immunoreactivity. Across all groups, astrocytes maintained an unactivated morphology and an astroglial response towards the implanted mNPC was never detected 16 weeks after implantation.

### Behavioral Assessment Immediately After Implantation

Survival, weight gain, and developmental milestones (DM) were assessed in all pups from postnatal day 2 (PND 2) to PND 15. Survival after the implant procedure and in the 48 hours post-implant was greater than 98%. Post-implant deaths were in smaller pups (<1.4 grams). The deceased pups were unable to be karyotyped because of maternal cannibalism. All surviving pups continued to have milk bands and robust vocalization during neonatal assessment in the days immediately following PND 2 implantation.

Surviving pups showed steady and typical weight gain over time for their karyotypes. Trisomic pups weighed significantly less than disomic pups at time of treatment (mean = 1.8 grams trisomic *vs.* 2.3 grams disomic pups, p<0.05) and throughout early development (F(13, 2158) = 5.92, p<0.001). Implantation of mNPC or saline did not affect weight gain in either karyotype through weaning, with implanted groups having similar weights as their respective untreated karyotype controls (F(26, 2158) = 0.50, p>0.05) (Figure S3).

The achievement of motor skill DMs (righting response, cliff avoidance, and negative geotaxis) occurred between PND 5 and PND 10, whereas eye and ear opening milestones emerged between PND 11–13. Occasionally, pups would have a mature motor skill response before treatment occurred on PND 2, and such pups were not included in the statistical analysis for that milestone. Thus, the number of litters and total number of pups contributing to each milestone differed (see Table S1 and Methods).

#### Righting Response

**Figure 6:**
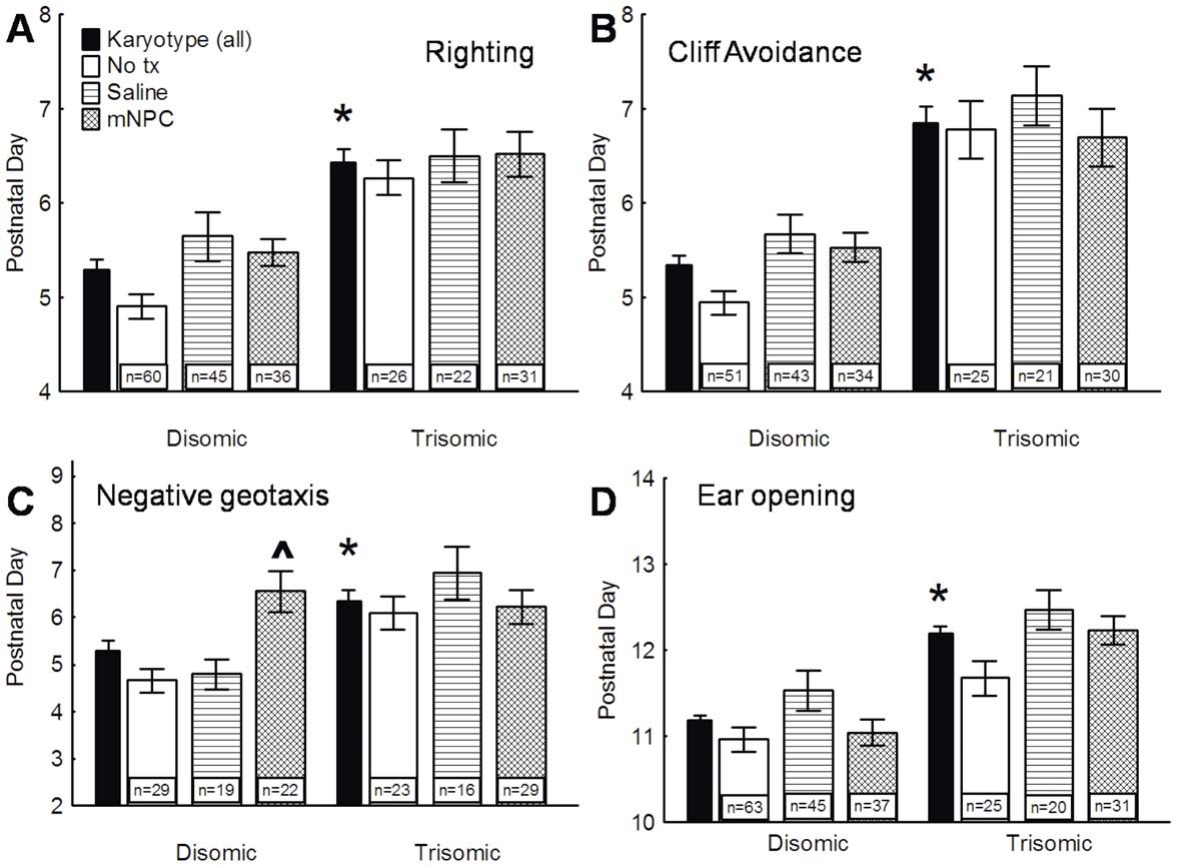
Trisomic pups were delayed in DMs and did not improve with treatment. A, B. Righting response (A) and the cliff avoidance (B) were delayed in trisomic pups and this delay was not altered by the implantation of saline or mNPC. C. Trisomic pups were also delayed in negative geotaxis. While trisomic pups were unaffected by treatment, the implantation of mNPC significantly delayed disomic pups to a degree that was comparable to the significant delays observed in the trisomic pups. D. Trisomic pups were delayed in time to ear canal opening. Treatment with both mNPC produced a delay in ear opening compared to untreated pups, while treatment with saline produced a significant additional delay. There was no interaction effect, indicating that both karyotypes were affected in a similar manner. * A significant main effect of karyotype indicates that overall trisomic pups were delayed compared to disomics. ∧ Indicates a significant delay due to the main effect of treatment regardless of karyotype (post hoc p<0.05). ‘n’ is the total number of pups in each group. Average day of achievement ± SEM shown.

The righting response is dependent on the coordination of muscles and detection of the supine position. A two-way ANOVA confirmed significant differences between karyotypes in the time to develop a mature righting response F(1, 214) = 34.123, p<0.0005) (Figure 6a). Disomics achieved the righting response 27.3 hours earlier than trisomics. Treatment with saline or mNPC did not significantly alter the righting response (F(2, 214) = 2.73, p>0.05), nor was there an interaction between the karyotype and treatment (F(2, 214) = 0.66, p>0.05).

#### Cliff Avoidance

Cliff avoidance relies on the detection of a short drop in front of the pup and the physical ability to turn away from the cliff. Analyses revealed a significant difference in the time to develop this ability between the disomic and trisomic pups (F(1, 198) = 56.88, p<0.0005) (Figure 6b). All disomic groups, regardless of treatment, achieved a developed response approximately 36 hours before any trisomic group. Again, neither implant type further delayed the development of cliff avoidance (F(2, 198) = 2.49, p>0.05), nor was there a significant interaction (F(2, 198) = 1.02, p>0.05).

#### Negative Geotaxis

Pups placed on an inclined plane with their nose pointing down will turn so that their nose points uphill. Again, trisomic pups were delayed in this DM by as much as 24 hours compared to disomic pups (F(1, 132) = 11.05, p<0.005) (Figure 6c). However, treatment with mNPC preferentially inhibited the disomic pups (F(2, 132) = 5.15, p<0.05). Disomic mice implanted with mNPC were delayed to a level that was indistinguishable from the trisomic groups (p>0.05) and significantly longer than either the untreated or saline disomic groups (p>0.05). In trisomic mice, no additional delays beyond the initial karyotype-induced delay were found (p<0.05), with no significant differences between the implanted and untreated groups.

#### Eye and Ear Opening

At birth, eyelids are shut, the ear pinnae (external ear flap) lay flat against the head, and ear canals are sealed. Events of neonatal development are eye opening, and the pinnae rise away from the head and the ear canal opening. In the present study, both eye and ear opening occurred later in development than the motor milestones, between 9 and 11 days *after* PND 2. A two-way ANOVA revealed that a significant difference in the time to eye opening existed between the two karyotypes, although the trisomic delay was only 19.5 hours (F(1, 215) = 66.31, p<0.0005). Treatment with either saline or mNPC produced no significant differences between disomic or trisomic mice, indicating that implants had no adverse effects on eye opening (F(2, 215) = 1.48, p>0.05), nor was the interaction significant (F(2, 215) = 0.60, p>0.05) (data not shown).

Results from the ear opening milestone indicated the same overall trisomic delay seen in the eye opening response, with a delay of 24.4 hours (F(1, 215) = 88.69), p<0.005) (Figure 6d). Unlike the eye opening response, treatment significantly delayed the time to ear canal opening (F(2, 215) = 12.82, p<0.0005). Treatment with mNPC produced a delay of 11.0 hours in comparison to untreated pups, with saline implantation producing an *additional* 6.7 hour delay (p<0.05). This treatment effect was consistent across both karyotypes, as indicated by an insignificant interaction (F(2, 215) = 2.23, p>0.05).

### Adult Behavior

#### Plus Maze

The Plus Maze is dependent on spontaneous exploration and measures the ability to remember previously visited locations. A cognitively intact animal will show greater exploration of arms not recently visited, resulting in increased novel alternations. Trisomic mice made an average of 5 more arm total entries than disomic mice (F(1, 73) = 4.36, p<0.05), although this was not affected by either treatment (F(2,73) = 0.22, p>0.05), nor was there an interaction (F(2,73) = 0.76, p>0.05). Because of this difference in total arm entries, data were normalized to the total number of possible novel alternations based on actual number of arm entries for each animal. Total arm entries ranged from an average of 30.2 arm entries in Disomic/mNPC mice to 40.4 in Trisomic/mNPC mice for the single 8 minute trial.

**Figure 7:**
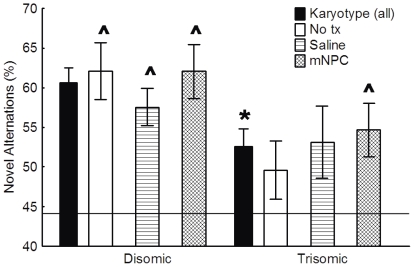
Novel alternations did not differ but mNPC treatment increased Trisomic performance above chance performance. Overall, trisomic mice showed significantly fewer novel alternations compared to disomic mice (* p<0.05). Treatment with either saline or mNPC did not significantly change the number of novel alternations in either karyotype compared to their respective controls. However, trisomic mice treated with mNPC alternated significantly above chance alone (solid line), while untreated and saline treated trisomic mice did not. Mean ± SEM shown. * Significant main effect of karyotype. ∧ Novel alternations were significantly above chance.

Chance performance, based on 4/5 novel arm entries, has been previously determined to be 44% [14]. A single sample T-test was used to determine if individual groups were performing significantly above this chance performance. All disomic groups alternated at a level significantly above chance, indicating purposeful exploration (p<0.0005 for each disomic group). Trisomic/No tx mice had a novel alternation rate of 49.6% which was not significantly above chance (t(8) = 1.46, p>0.05). Trisomic/Saline mice also did not alternate above chance (t(8) = 1.90, p>0.05). However, the Trisomic/mNPC group, with an alternation rate of 62.1%, did alternate significantly above chance (t(12) = 3.82, p<0.005) indicating that this group, like the disomic groups, had a purposeful alternation approach (Figure 7).

Although some groups performed above chance, a two-way ANOVA was used to determine if there were significant differences in the overall number of novel alterations. In the percentage of *novel* alternations, there was a main effect of karyotype, with trisomic mice in general making fewer novel alternations than disomic mice (F(1,73) = 6.17, p<0.05) (Figure 7). Treatment with saline or mNPC did not significantly change the percentage of novel alternations (F(2,73) = 0.32, p>0.05), nor was there an interaction between karyotype and treatment (F(2,73) = 0.53, p>0.05). Thus, while the Trisomic/mNPC group performed above chance, the total number of novel alternations was not significantly different from the other trisomic groups.

#### Morris Water Maze (MWM) - Cued Platform and Swim Speeds

A cued platform session on Day 1 of the MWM swim task was used to determine motivation and swimming capabilities between all groups. Four trials of the visible platform task were administered on Day 1. Using a repeated measures three-way ANOVA, it was found that the trisomic groups had longer escape latencies overall compared to the disomic groups (F(1,62) = 9.60, p<0.005). However, the rate at which latencies shortened over the four trials was not significantly different (F(3, 186) = 0.60, p>0.05), indicating that animals of both karyotypes were motivated to perform the task (Figure S4a). Treatment with saline or mNPC did not produce significant effects on motivation (F(2, 62) = 0.19, p>0.05) or a significant interaction (F(6, 186) = 0.68, p>0.05). Analysis of swim speeds indicated that there were no significant differences in the speed at which animals swam in the MWM as a result of karyotype (F(1,40) = 0.05, p>0.05), treatment (F(2, 40) = 0.89, p>0.05), or as an interaction between the two variables (F(2, 40) = 0.20, p>0.05). Thus, differences in latencies measured during the hidden platform task on Days 2–7 reflect changes in learning and not in motivation or swimming abilities.

#### Morris Water Maze - Hidden Platform

**Figure 8:**
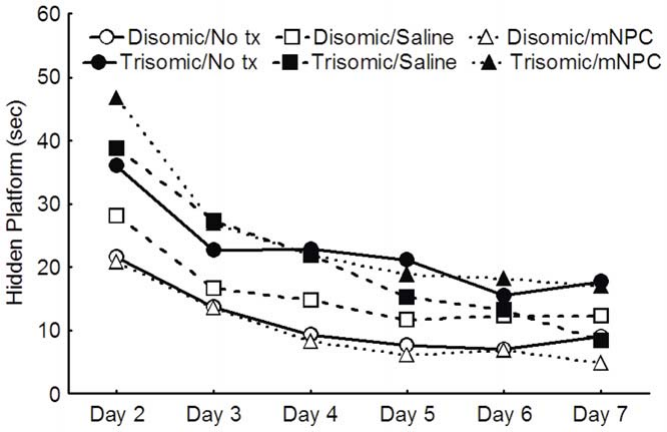
In the MWM, all groups improved hidden platform task performance at a similar rate. All groups had decreasing latencies across the 6 days of hidden platform testing, as indicated by the parallel slopes of the lines; however, the trisomic groups were consistently slower than the disomic groups. Treatment did not affect the latency to the hidden platform in either karyotype.

On Days 2–7 of the MWM, mice were trained in the spatial portion of the maze in which the platform is hidden under one cm of opaque water. A repeated measures three-way ANOVA revealed significant karyotype differences across the daily average latency (F(5,310) = 3.64, p<0.05). Disomic mice, regardless of treatment, significantly decreased their latency between Day 2 and Day 3 (p<0.05), but then slowed to an asymptotic level of performance on Days 3–7 (p>0.05), suggesting that this group successfully learned the task after two days of training (Figure 8). By comparison, the trisomic groups did not reach an asymptotic level of performance until Day 5, as indicated by no significant decreases in latency between Days 5–7 (p>0.05) (Figure 8). Treatment with either saline or mNPC did not affect the ability of either karyotype of mice to learn the location of the hidden platform (F(10, 310) = 0.99, p>0.05).

In the probe trial, the hidden platform was removed and the number of times the animal crossed the former platform site was recorded. Trisomic mice overall had significantly fewer platform crossings than disomic mice (F(1,61) = 4.36, p<0.05). Treatment had no significant effect on the number of platform crossings (F(2, 61) = 0.15, p>0.05) (Figure S4b).

#### Conditioned Taste Avoidance (CTA)

CTA is a classical conditioning task, in which animals must learn the association between a novel stimulus (chocolate milk, CS) and an unconditioned aversive response (nausea). Cognitively intact animals will reduce their consumption of the CS on second exposure. Previous work in our laboratory has shown that trisomic Ts65Dn mice are impaired in their ability to form a CTA, and that this test can be used as a screen for novel treatments designed to improve cognition in DS (K.N. Maclean, unpublished data).

Average daily water consumption on training days 2–5 did not differ between the two karyotypes (F(1, 90) = 0.16), p>0.05) or as a result of treatment (F(2,90) = 1.98, p>0.05). All groups consumed between 1.3 and 1.6 mLs of water a day. The amount of CS consumed on first exposure (Training Day) was not affected by either karyotype (F(1,90) = 2.37, p>0.05) or treatment (F(2,90) = 0.95, p>0.05), or as a function of the interaction between karyotype and treatment (F2,90) = 0.28, p>0.05), indicating that all groups preferred the CS to water (Figure S5). Consumption levels for each group exceeded that of normal water consumption by more than 35%. Individual mice that had a greater than 10% avoidance of the CS on first exposure compared to normal water drinking were identified as neophobic (n = 12). These animals were excluded from further CTA analyses (Neophobic mice by group: Disomic/No tx n = 3; Disomic/Saline n = 2; Disomic/mNPC n = 0; Trisomic/No tx n = 2; Trisomic/Saline n = 1; Trisomic/mNPC n = 4).

**Figure 9:**
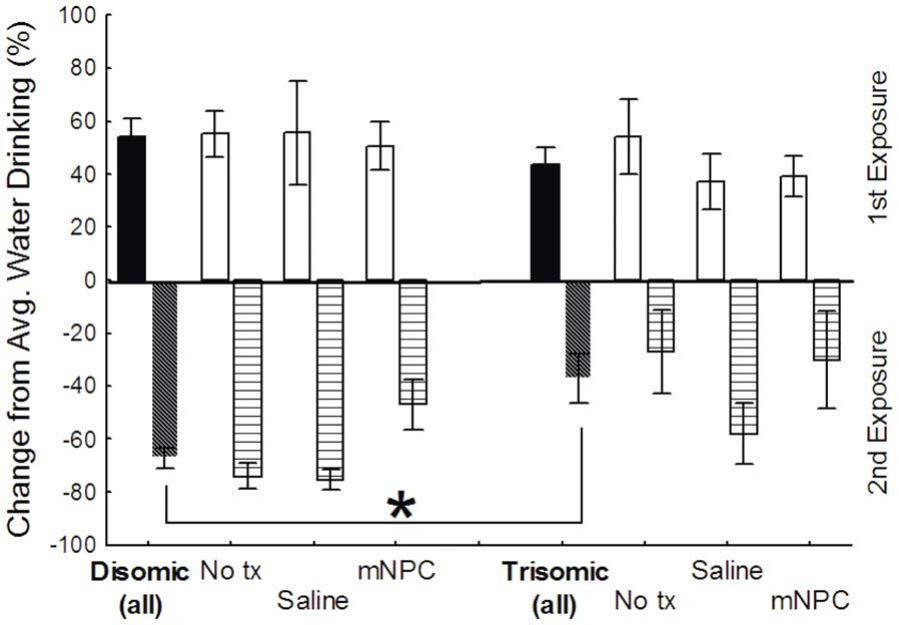
Trisomic mice continued to drink significantly more CS on second exposure than disomic mice. There was no significant difference in degree of preference during first exposure between disomic and trisomic animals. During the second exposure disomic mice had a greater avoidance of the CS than the trisomic animals (* p<0.05). Treatment did not preferentially affect CTA learning and affected both karyotypes to the same degree. Mean ± SEM shown.

The difference in CS consumption between first and second exposure indicated that trisomic mice did not avoid the CS on second exposure to the same degree as disomic mice (F(1,90) = 8.36, p<0.005). As reported above, the first experience with the CS induced an increase in CS consumption in both karyotypes by more than 35%. During the second experience with the CS, the disomic mice reduced their CS drinking by more than 60% on the second exposure (Figure 9). This decrease was significantly greater than the change measured in trisomic mice, which as a group had less than a 40% reduction (p<0.05), suggesting the association between the CS and nausea was not as strongly learned in the trisomic mice. Treatment with saline or mNPC did not affect CS consumption on second exposure (F(2,90) = 1.72, p>0.05), nor was there an interaction of karyotype and treatment (F(2,90) = 0.24, p>0.05).

#### Novel Object Recognition (NOR)

Novel object recognition relies on memory for familiar objects and the preference to explore novel objects. A repeated measures three-way ANOVA comparing total exploration time, irrespective of which object, indicated that there were no significant differences in the general exploration behavior as a function of karyotype (F(1,26) = 0.08, p>0.05), treatment (F(2,26) = 0.12, p>0.05), or their interaction (F(2, 26) = 0.318, p>0.05)(Figure S6). Exploration behavior was highly variable between individual mice, with some mice only exploring 15 seconds and other spending as much as 90 seconds. To verify that the ability to remember a familiar object was not dependent on the amount of time mice initially spent exploring it, a Pearson's r moment correlation was used to determine that the time exploring during Exposure 1 was not significantly correlated to individual memory data taken during Exposure 2 (Pearson's r = 0.02, n = 32).

**Figure 10:**
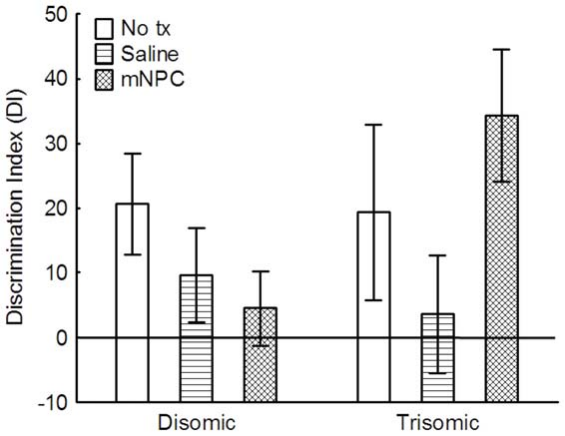
All groups recognized the novel object. All groups exhibited a preference for the novel object, as indicated by a DI above zero. While there is apparent variability, the differences in the DI between groups did not reach significance (p>0.05). Mean ± SEM shown.

Memory for a familiar object, and thus the ability to recognize a novel objects, was determined using a discrimination index (DI). If both objects were explored equally, then the DI would approximate zero. When a novel object is explored more, it is indicated by a positive DI suggesting a better memory for familiar objects. All groups had an average DI measure above zero. A two-way ANOVA indicated that across groups, the DI measures were not significantly different as a function of karyotype (F(1,26) = 0.77, p>0.05), treatment (F(2,26) = 1.13, p>0.05), or as function of their interaction (F(2,26) = 1.63, p>0.05) (Figure 10). Thus, trisomic mice were not impaired in this task, and explored the novel objects to a similar degree as the disomic controls. Further, treatment did not alter the time spent exploring novel objects.

## Discussion

This study was designed to investigate the effects of a neonatal mNPC intervention on the cognitive and neuroanatomical changes that occur in DS, compared to sham saline and untreated controls. The implantation of mNPC may not be a viable early intervention for DS at this time. Despite evidence of some long-term survival, there were no reliable improvements in cognitive function.

Both the trisomic and disomic neural environments allowed for the survival of implanted mNPC throughout the changes associated with neonatal development, adolescence, and adulthood. While the rate of survival was low, the proportion of animals with surviving implanted cells was higher than those previously reported [30]. Further, a greater proportion of trisomic brains had surviving cells than disomic brains, even though the disomic brains had greater total numbers surviving and greater migration. Although the brain is a partially immunologically privileged site, the limited survival of implanted cells remains a significant challenge in the field of cell transplantation research. We had hypothesized that by implanting at PND2, we might overcome the low post-implantation survival rate of NPC reported in many adult implant studies [31], [32]. At this time point, we were able to prevent the formation of a glial scar, another limitation to effective transplantation, but did not increase the number of surviving implanted cells compared to these previous studies.

Earlier studies suggest that implanted NPC migrate to sites of damage in the injured brain by following chemokine signals [33], [34]. The presence of implanted mNPC in certain areas of the brain may indicate a need for support in those areas. In the current study, we found that the implanted mNPC had migrated throughout the brain in both karyotypes. However, trisomic brains contained more mNPC at the site of implantation, the hippocampus, than did disomic brains. This may indicate that the hippocampus in trisomic mice is especially compromised, but not enough mNPC survived to affect cognition. Alternatively, the low migration rate may suggest that implanted mNPC did not or were unable to migrate readily in the trisomic host environment.

One mechanism we investigated for producing long-term change in the trisomic brain was the potential for mNPC to decrease the DG hypocellularity reported in Ts65Dn mice [16], [19], [35]. A karyotype specific hypocellularity was not found in this study. This could result from differences in quantification methods or, more likely, the postnatal handling during DM assessment, which can increase the retention of immature and mature DG neurons [16], [19], [36], [37]. Despite the lack of karyotype controlled changes, implantation with mNPC, but not saline, resulted in an increased density of dentate granule cells. We did not find evidence that implanted mNPC differentiated into granule cells, as the total number of mNPC found in this area could not account for the 33% increase in granule density measured in implanted animals. This would suggest that the effects of the mNPC are indirect, facilitating an environment that promotes endogenous DG neurogenesis, a phenomenon that has been previously reported in the aged hippocampus implanted with NPC [32]. This ability to increase the density of the DG may have beneficial effects in older Ts65Dn mice, which also have fewer neurons in the DG [16].

No significant effects of mNPC implantation on cognition were found. It is possible that the low number of surviving cells was not enough to affect cognition. While some individual animals that received implantations of either saline or mNPC had impressive performances in some behavior tasks, the effects were not consistent within karyotypes, treatments, or across tasks. The limited survival of implanted cells precluded statistically meaningful correlations with behavior data, and the degree to which survival corresponded with improved performance on behavioral tasks was inconsistent.

### Concluding Remarks

The current study confirmed the cognitive deficits of trisomic Ts65Dn mice compared to disomic mice in Developmental Milestones, Plus Maze, MWM, and CTA measures of cognition. The treatment with mNPC did not enhance the cognitive function of either trisomic or disomic mice. Low surviving numbers of mNPC and modest changes in granule cell density may not have been sufficient to produce changes in behavior. However, while the implanted mNPC did not affect the cognitive abilities of the trisomic mice in adulthood, it is possible they will offer some protection against the secondary cognitive decline of Alzheimer's disease that emerges in DS [38], [39]. The modest improvement in granule cell density and novel alternations in the Plus maze could be suggestive of differences that will be significant with time. These results contribute to a growing foundation of information on developmental delays and cognitive impairments in the Ts65Dn mouse, confirming the fidelity of this model with previous studies and providing insight into possible alternative treatment innovations in the future.

## Methods

### Ethics Statement

All animal manipulations and housing were in accordance with the National Institutes of Health Guidelines for the Care and Use of Laboratory Animals. Protocols were approved by the Institutional Animal Care and Use Committee at the University of Colorado Denver which holds the following permits and accreditations: American Association for Accreditation of Laboratory Animal Care (AAALAC; file 00235), PHS Animal Welfare Assurance of Compliance (number A3269-01), USDA License (#84-R-0059). Neonatal implantation and adult euthanasia were done following the guidelines for age appropriate anesthesia and euthanasia. Every effort was made to minimize pain and distress.

### Animals and Husbandry

The Ts65Dn mouse is a commonly used model for studying cognitive impairment in DS [40]–[43]. Approximately 25% of pups in a litter express a partial triplication of mouse chromosome 16, which contains 60% of the genes human chromosome 21 [11], [44]. Disomic littermates provided karyotype controls. Monogamous breeding pairs of trisomic Ts65Dn females (Jackson labs stock #001924) and C57Bl/6JEixC3H/HeJ F1 generation disomic males were housed in a temperature/humidity controlled room on a reverse 12-hour light/dark cycle. The colony was propagated using the Ts65Dn female offspring from the original pairings and C57Bl/6JEixC3H/HeJ F1 generation disomic males (offspring of Jackson labs female C57/Bl/6JEi Jax #JR 0924 and male C3H/HeJ Jax #JR 000659 pairings). Dams were monitored daily for new litters and day of birth was defined as PND 0. On PND 2, all pups in a litter were implanted with mNPC (n = 16 litters) or saline (n = 20 litters) as described below. Additional litters (n = 16) remained unimplanted, as controls for the implant procedure. Pups were weaned, separated by gender into groups of 3–5, and karyotyped by fluorescent in-situ hybridization (FISH) using blood smears derived from tail snips as previously described on PND 21 [17]. Only male mice were used in behavior studies at 16 weeks, as females were used for breeding purposes. Mice with the retinal degeneration mutation Pde6b(rd1), which results in blindness, were identified by examination with an indirect ophthalmoscope. These mice were excluded from all adult behavior studies except CTA. Food and water were provided *ad libitum*, *e*xcept during the CTA.

### Neural Progenitor Cells - C17.2 mNPC line

The C17.2 mNPC line (provided by Dr. Evan Snyder, Sanford-Burnham Medical Research Institute) is a clonal multipotent disomic NPC line derived from postnatal male mouse cerebellum, immortalized with *v-myc* [30], [45] and stably transfected with green fluorescent protein (GFP). The cell line has been characterized as a true progenitor cell line, capable of differentiation into both neural and glial cells, migration to sites of damage, synaptic integration with host tissue, and secretion of growth factors [20], [22], [25], [46]–[52]. As C17.2 cells are committed to a CNS lineage, their tumorogenicity is low [30]. This immortalized cell line possesses the qualities of undifferentiated NPC, making it suitable for transplantation studies.

All C17.2 mNPC were maintained as an undifferentiated monolayer on 10 cm^2^ tissue culture treated polystyrene plates. C17.2 mNPC were grown in highly modified DMEM feeding medium (Dulbecco's minimal essential medium - Gibco cat.10-013-CV) with 10% fetal bovine serum, 5% horse serum, 2 mM glutamine, 100 IU/mL penicillin, and 100 µg streptomycin/mL. All cell culture chemicals were purchased from Invitrogen Life Technologies unless otherwise indicated. Half of the media was changed every 2–3 days and cells were split (1∶10) weekly, except when mNPC were prepared for implantation.

For mNPC implant preparation, 90–95% confluent plates were split (1∶5) 48 hours before being used. C17.2 cells were *not* pre-differentiated toward a specific neural fate prior to implant. On implantation day, mNPC were rinsed three times with sterile Hanks Balanced Salt Solution (HBSS), dissociated using Trypsin-EDTA (1.5 mL/plate, 0.05%). Cells were concentrated to 100,000 viable cells/µl in sterile 0.09% saline. Trypan Blue exclusion determined cell viability was >85% in all implant preparations. Viability counts of mNPC remaining after implant were similar to counts obtained prior to implant, with only a ±2% maximum difference found.

### Implantation Procedure

All disomic and trisomic littermates received the same implant treatment two days after birth (PND 2). On PND 2, the dam was separated into a holding cage, and returned to the home cage once all pups were treated. The separation period averaged 20 minutes and was always less than 45 minutes, depending on litter size. During this time, baseline weights and developmental milestones were taken for each pup.

Pups were cryoanesthetized until non-responsive to stimuli, then implanted bilaterally with 100,000 undifferentiated C17.2-GFP mNPC (1 µl) or with 1 µl sterile 0.09% saline. Freehand injections in the dorsal hippocampus (+2 mm AP, ±1 mm LM, −2.5 mm DV from *lambda*) were performed using a Hamilton syringe with a 30-gauge needle. The neuroanatomical point *lambda* (as opposed to *bregma*) was used as a reference point, because the overlying blood vessels at *lambda* are visible through the skin on PND 2. All implants were made through the undisturbed skin and skull to minimize trauma and risk of infection, since the skull is still pliable. Litters not receiving an implant were removed from the dam for a period similar the implanted litters and returned to the home cage without implantation.

### Tissue Preparation and Immunohistochemistry

Adult mice were anesthetized with an intraperitoneal injection of sodium pentobarbital (60 mg/kg) and were perfused intracardially with 0.9% saline followed by fixation with fresh, ice-cold 4% paraformaldehyde (PFA) for 10 minutes each. Brains then were removed and stored in fresh 4% PFA overnight at 4°C, then cryoprotected in a 30% sucrose until they sank, usually 48–72 hours later. Brains then were embedded in Optimal Cutting Temperature medium (Tissue Tek, Fisher 14-373-65), sectioned as freefloating coronal sections (40 µm), and stored at −20°C.

Immunohistochemistry (IHC) was used to identify the survival of the mNPC in the host brains using anti-GFP (Abcam 290, 1∶50K (5 mg/mL), 72 hour incubation). Double-label was done using anti-GFP and anti-MAP2 (neurons, Chemicon AB5622, 1∶500, (1 mg/mL)), anti-GFAP (astrocytes, Millipore MAB360, 1∶500, (1 mg/mL)), and anti-reelin (hippocampal interneurons, Millipore MAB5364, 1∶1000, (1 mg/mL)). Single labeling against anti-GFAP and anti-Iba1 (Microglia, Wako STK 4406, 1∶500, (50 µg/1 mL)) was used to evaluate the glial response. Negative controls for each antibody included sections from brains without mNPC (i.e. saline and untreated), as well as the omission of the primary antibody in mNPC implanted brain sections.

Briefly, sections were rinsed of cryoprotectant, exposed sequentially to 0.3% peroxidase block, 5% goat serum protein block, a biotin/avidin block, and placed in primary antibody. Sections were incubated overnight at 4°C for all primary antibodies except anti-GFP which was incubated for 72 hours. After incubation in primary antibody, sections were rinsed and incubated in a biotinylated secondary antibody or a fluorescent secondary antibody if double labeling procedures were used. Biotinylated antibodies were visualized with a 3,3′-diaminobenzidine (DAB)-nickel sulfate reaction. After staining, sections were mounted onto glass slides and coverslipped with Permount. Fluorescent sections were mounted immediately after rinsing the secondary antibody and coverslipped using anti-fade mounting medium.

A subset of animals that underwent behavior testing were used for pilot studies to determine immunohistochemical and quantification parameters; these animals are not included in the results. GFP+ mNPC survival was determined from brain sections taken every 480 µm and spanning the rostral-caudal range of the brain. GFP+ cells were never found outside −0.26 to 3.0 mm relative to bregma, thus only sections within this range were used for quantifying mNPC survival. Extrapolations using the number of GFP+ cells weighted by the number of sections in the rostral-caudal range were used to estimate the percent total surviving cells [51], [53]. A Student's t-test was used to identify significant differences between the karyotypes in the total number of surviving mNPC and the number of mNPC in the hippocampus. Cell counts were weighted by the total number of sections stained for GFP. Double labeling to determine mNPC cell fate was performed on sections taken every 480 µm. Sections taken every 240 µm for GFAP+ astrocytes and every 480 µm for Iba1+ microglia were analyzed to determine if a glial scar resulted from the neonatal implantation procedure.

### Cresyl Violet Staining and Stereology

Quantification of granule cells of the DG was performed on every 6th section of tissue, approximately every 240 µm. Unstained sections were mounted, allowed to air dry, rehydrated in a decreasing series of alcohols, and placed in cresyl violet staining solution (0.25%). Three sections containing the dorsal hippocampus (AP-1.70 to −2.30 mm) were used from each brain. Nine photomicrographs (20× magnification) were taken of the granule layer of the DG for quantification in each section. The diameters of fifteen cells in each blade of the DG were measured in the most rostral section used to determine average cell diameter.

Unbiased stereological methods, based on a modified fractionator method, were used to quantify the number of neurons per mm^2^ area [54]. For each photomicrograph, a 4×4 grid was overlaid and neurons were counted in every other square. Cells crossing the left and/or bottom border of a square were excluded. The areas of the neuronal layers also were measured from these photos using the Digimizer Software. The error coefficient (CE(N)) was calculated as previously described [54] and was found to be less than 0.10, indicating the number of sections and the total area counted was sufficient to obtain reliable counts. All hippocampal analyses were made by a researcher blinded to the karyotype and treatment. Ten percent of sections were counted by a two additional researchers, who also were blinded to karyotype and treatment. The average correlation between raters was r = 0.94 and the concordance between raters was found using Kendall's coefficient of concordance = 0.51, indicating that the counts obtained by all researchers were similar.

A two-way ANOVA was used to determine density differences between groups. Significance was established at p≤0.05 and when appropriate Fisher's least significant difference (LSD) *post-hoc* comparisons were used.

### Behavior Testing Methods: Neonatal Behavior Studies

#### Weight and Developmental Milestone (DM) Assessment

Prior to implantation on PND 2, baseline weights and five DMs were assessed: righting response, cliff avoidance behavior, negative geotaxis response, and eye and ear opening. DM assessment was performed in the same order every day and assessed similarly to those previously described methods [12], [55], [56]. All pups, regardless of sex, were included in the neonatal behavioral studies. DMs were measured with the following scoring system: ‘0’ - no response, ‘1’ - attempted or partial response, and ‘2’ – a complete, developed response. DM achievement was monitored until the milestone was performed successfully for two of three consecutive days. The first day of the three consecutive days with a score of ‘2’ was recorded as the day of achievement and used in analyses. Pups that could perform a task during baseline measures on PND 2 were excluded from analysis for that particular milestone, as the achievement of the DM occurred *before* the implantation procedure on PND 2 and thus its emergence could not be an effect of treatment. For this reason, the number of pups included in each milestone varied (Table S1).

Baseline and subsequent postnatal weights were analyzed using a repeated measures ANOVA, with the following variables: karyotype (trisomic vs. disomic), treatment (mNPC vs. saline vs. untreated), and days (PND 2 – PND 15) as the repeated measure. A Fisher's least significant difference (LSD) *post hoc* test was applied when significant main effects or interaction effects were found (p≤0.05). Weights data were reported as average number of grams ± SEM for each day.

DM data were analyzed using a two-way ANOVA in which data was the average day of achievement of the karyotype group for the litter rather than individual pups. Analyzing the data by litter, rather than by individual pups, prevents a confounding effect of litter size, mothering, and within-litter and between-litter differences that occur in motor development [57]–[59]. Each litter mean was weighted by the number of pups in the litter with the appropriate karyotype designation. Weighted litter means then were used in statistical analyses. Differences resulting from karyotype or treatment were determined using a two-way ANOVA for each DM, with significant differences at p≤0.05 and a Fisher's LSD *post hoc* test used when appropriate. DM data were reported as mean day of achievement ± SEM.

### Behavior Testing Methods: Methods for Adult Behavior Studies

After weaning, male mice were group housed, but tested individually for behavior. Mice in the same home cage always were tested in the same order, which occurred during the initial dark hours of the light/dark cycle. Mice that were positive for retinal degeneration were excluded from the Plus Maze, Morris Water Maze, and Novel Object Recognition tasks; neophobic mice were excluded from CTA. Thus, the number of animals used in each study varied (Table S1).

#### Plus Maze (PM)

The Plus Maze assesses a mouse's memory for recently visited maze arms based on spontaneous alternation. Rodents inherently choose to explore more novel environmental stimuli, resulting in ‘novel alternations’ [60]. Ts65Dn mice have a significantly lower rate of novel alternation in the PM as compared to disomic control mice at 4 months of age [14]. This test can also be used as a measure of anxiety, so to avoid confounding the cognitive results with measures of anxiety, the maze was not elevated and the four arms were enclosed with clear plexiglass walls with a white floor, similar to the procedure outlined in [14]. Each arm measured 3 inches wide, 18 inches in length, and had a black and white visual cue at the end.

In this one trial task, mice were allowed to explore a plus shaped maze for eight minutes. All sessions were recorded digitally and manually analyzed for novel alternations, defined as entering four novel arms within five arm entries. An entry was recorded when all four feet of the mouse crossed the threshold of the arm. Chance performance was defined previously as 44% [14]. Learning in this task was expressed as percentage of novel alternations normalized to the number of possible alternations, based in the number of arm entries. Total number of arm entries and novel alternations were analyzed using two-way ANOVAs and Fisher's LSD *post-hoc* test when appropriate. Significant deviations from chance performance were determined using single sample t-tests for each karyotype/treatment group.

#### Morris Water Maze (MWM)

The MWM depends on spatial memory to allow a mouse to find a platform below the water level in opaque water. The task was divided in three parts. First, a single session of four trials determined swim speed and task motivation using a submerged platform cued with a flag (visible platform) (Day 1). Visible platform latencies were analyzed using a repeated measures three-way ANOVA. Spatial memory was measured using a hidden platform task in which the platform is 1 cm below water line without a visual cue above water. Average latency for each of the six days (Day 2–7) of the hidden platform task (4 trials/day) again were analyzed using a repeated measures three-way ANOVA, with daily average latency being the repeated measure. A final probe trial was completed after the four hidden platform trials on Day 7. In this trial, the platform was removed and the mouse was allowed to swim feely for 60 seconds. The number of platform crossings and swim speeds were analyzed using a two-way ANOVA and Fisher's LSD *post hoc* test.

The MWM was performed in a 34-inch diameter circular pool filled with water made opaque by the addition of non-toxic white poster paint. Water temperature was 19°C±1°C. Four visual cues were affixed along the walls of the pool and were associated with four quadrants. Animals were placed in the water facing a visual cue, in one of the three quadrants that did not contain the platform. The original visible platform position (Day 1) was changed in the hidden platform task (Days 2–7). The platform remained in the same position for all days of the hidden platform task. The animal's starting quadrant was randomly determined and repeated for each mouse in the session. Trials were separated by 13 minutes, with mice being placed in a dry cage under a heating lamp to prevent the potentially confounding effects of hypothermia [61]. One session of four trials occurred daily, and time to reach the platform was recorded. If at the end of the 60 seconds the mouse had not found the platform, he was guided to it. Animals were allowed 30 seconds on the platform to formulate a cognitive map in reference to the four visual cues. All sessions were digitally recorded and analyzed both manually (latency, platform crossings) and using AnyMaze Software (swim speeds).

#### Conditioned Taste Avoidance (CTA)

CTA is a classical conditioning task where a novel flavor (the conditioned stimulus, CS) is paired with a feeling of nausea, produced by an injection of lithium chloride (the unconditioned stimulus, US). The association between the CS and the US induces an avoidance of the novel flavor on subsequent exposures [62]. CTA was administered as a two-bottle paradigm over 8 days. Mice were trained to first drink their fill of water during 60 minutes each day, then were given an intraperitoneal (IP) injection of 0.9% sterile saline (0.0075 ml/g) and returned to their group-housing cage. The amount of water consumed from each bottle was recorded. On Day 6 (Training Day), both bottles were filled with fat-free chocolate milk, the first exposure to the CS. After the 60-minute drinking period, mice were given an IP injection of 0.4 M lithium chloride (0.0075 ml/g). After the injection, mice were monitored for signs of nausea. The amount of CS consumed from both bottles was recorded. On Day 8 (Test Day), mice were offered one bottle of chocolate milk (second exposure) and one bottle of water. The amount consumed from each was recorded.

To characterize and measure CTA, several statistical analyses were done. A two-way ANOVA compared average water consumption (Days 2–5) between groups to verify that all groups drank same amount of water. To validate that all groups had a similar preference for the CS and to eliminate individual mice with a neophobic response, the amount of chocolate milk drank on first exposure (Day 6) was normalized to the average water consumption on Days 2–5. Mice with a neophobic response were excluded from further CTA analyses, as determined by a greater than 10% decrease in chocolate milk intake on first exposure (Day 6) compared to the average amount of water that was consumed on Days 2–5. Learning was analyzed with a two-way ANOVA comparing the percent change in normalized CS consumed between first exposure and second exposure.

#### Novel Object Recognition (NOR)

Cognitively intact animals will spend more time exploring a novel object over a familiar object, however trisomic mice have been reported to be deficient in novel object recognition [63]. A simple discrimination test paradigm was administered similar to that as described in [64]. Briefly, mice were allowed to explore two identical objects for 15 minutes during Exposure 1. On Exposure 2, 24 hours later, animals explored a familiar object from Exposure 1 and a novel object. All sessions were recorded for subsequent manual analysis. To verify that all groups spent the same amount of time exploring, the total exploration time (minutes) was analyzed using a repeated measures three-way ANOVA, with Exposure 1 and Exposure 2 being the repeated measures. A Pearson's correlation was used to determine if learning was a function of the amount of exploration. Learning was measured based on the total time spent exploring the novel object on Exposure 2, compared to the total time spent exploring the familiar object on Exposure 2, resulting in a normalized discrimination index (DI: *Novel Object Exploration/Total Exploration – Familiar Object Exploration//Total Exploration*) [18], [64]. A DI of zero indicated that both objects are explored equally; a positive DI indicated that the novel object was explored preferentially. A two-way ANOVA was used to analyze the DI measures.

## Supporting Information

Figure S1 **Microglial presence in the hippocampus did not change with mNPC implantation.** Resting Iba1+ microglia were spaced evenly throughout the hippocampus in untreated disomic (A) and untreated trisomic (C) brains. The same pattern of distribution was seen in Disomic/mNPC (B) and Trisomic/mNPC (D). (B) and (D) were double labeled for Iba1 and GFP, resulting in higher background. As expected, no microglia co-labeled for GFP, indicating that implanted mNPC were not differentiating into a macrophage lineage. A1 is a magnification of boxed area in A to illustrate the resting morphology of the microglia. Scale bars in A–D = 500 µm.
(TIF)

Figure S2 **Astrocyte presence in the hippocampus of 16 week old brains revealed no gliotic scarring.** Disomic/No tx (A) and Trisomic/No tx (C) mice had ubiquitous and evenly spaced GFAP+ astrocytes, which did not appear to have an activated morphology. No brain sections were found that had a condensation of reactive astrocytes suggestive of a gliotic scar. No differences in the pattern of GFAP+ staining was observed in Disomic/mNPC (B) or Trisomic/mNPC (D) mice. Gr, granule cell layer; rad, stratum radiatum; CC, Corpus callosum. Scale Bars in A–D = 500 µm.
(TIF)

Figure S3 **Treatment did not alter weight gain in pups.** Typical for trisomic pups, their weights were lower than their disomic littermates (p<0.001). The implantation of saline or mNPC did not alter the weight gain of either group (p>0.05). Mean weight values of each karyotype/treatment group of pups are shown. *Significantly different weight at PND 21 between all trisomic and all disomic groups (main effect of karyotype). ↓ indicates day of treatment.
(TIF)

Figure S4 **Visible platform training and platform crossings were similar between all groups in the MWM.** A. Trisomic mice as a group had longer average latencies to reach the visible platform, however all groups improved their performances over the four trials, as indicated by the parallel lines. B. In the probe trial, all groups had a similar number of platform crossings. No group showed an increased tendency to perseverate in the platform location. Mean ± SEM shown.
(TIF)

Figure S5 **Both disomic and trisomic mice consumed similar volumes of CS on Training Day.** There was no significant difference in the amount (mLs) of CS consumed by either karyotype or treatment group on first exposure (p>0.05). Data subsequently was normalized to the average drinking behavior for each animal to account for individual variations.
(TIF)

Figure S6 **All groups had similar total exploration times in the NOR.** There was large intragroup variability on both days of exploration, especially for No treatment and Saline treated groups of both karyotypes. All groups explored significantly less on Day 2. Mean ± SEM shown.
(TIF)

Table S1 **Number of animals used in each cognitive test.**
(TIF)
