# Supplementary figures and images for: Effects of Neonatal Neural Progenitor Cell Implantation on Adult Neuroanatomy and Cognition in the Ts65Dn Model of Down Syndrome
Source: PLoS One. 2012 Apr 25;7(4):e36082. doi: 10.1371/journal.pone.0036082 (PMC3338504; doi:10.1371/journal.pone.0036082)

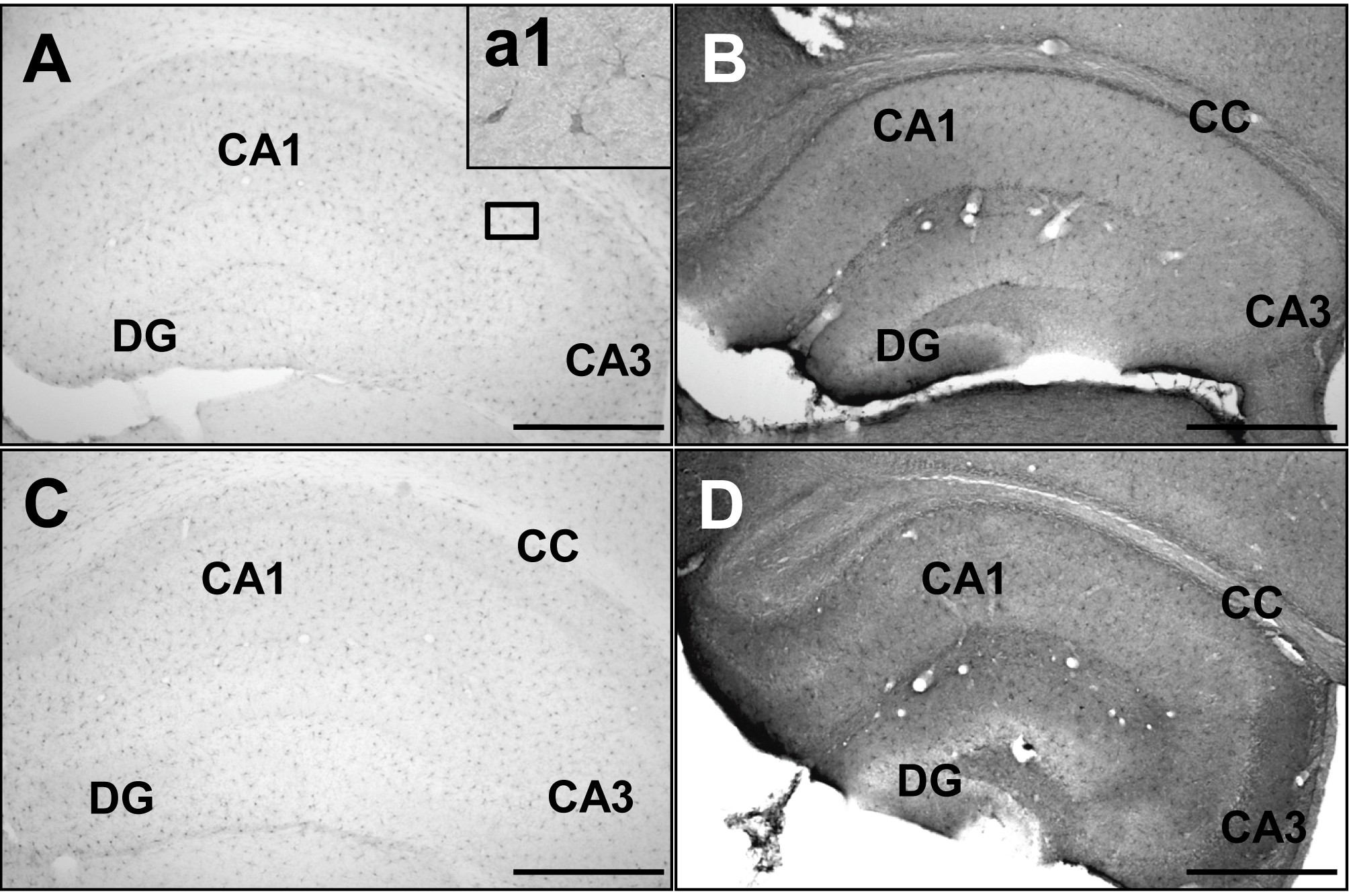

Supplement: Figure S1 — Microglial presence in the hippocampus did not change with mNPC implantation. Resting Iba1+ microglia were spaced evenly throughout the hippocampus in untreated disomic (A) and untreated trisomic (C) brains. The same pattern of distribution was seen in Disomic/mNPC (B) and Trisomic/mNPC (D). (B) and (D) were double labeled for Iba1 and GFP, resulting in higher background. As expected, no microglia co-labeled for GFP, indicating that implanted mNPC were not differentiating into a macrophage lineage. A1 is a magnification of boxed area in A to illustrate the resting morphology of the microglia. Scale bars in A–D = 500 µm. (TIF) [file pone.0036082.s001.tif]

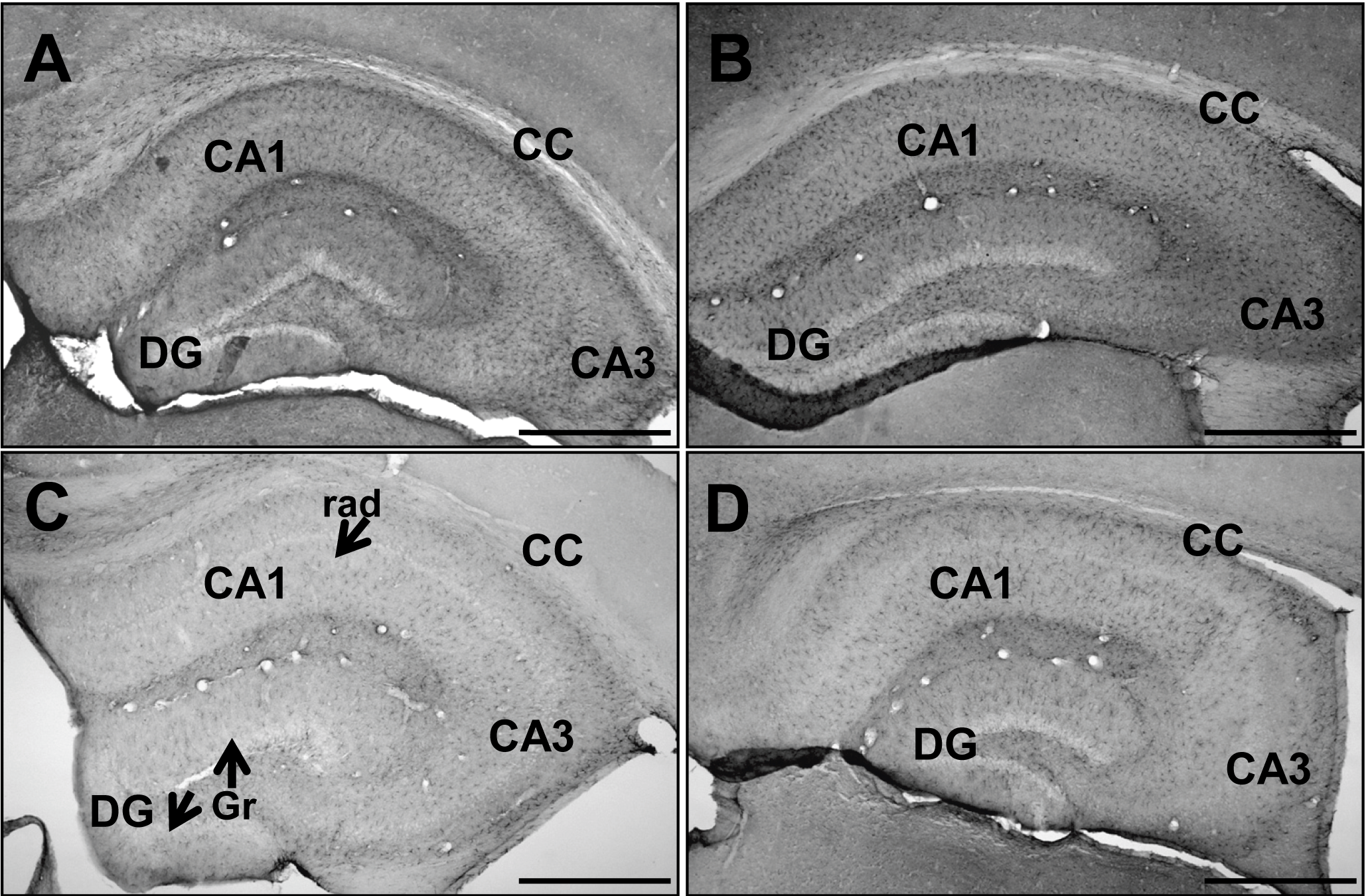

Supplement: Figure S2 — Astrocyte presence in the hippocampus of 16 week old brains revealed no gliotic scarring. Disomic/No tx (A) and Trisomic/No tx (C) mice had ubiquitous and evenly spaced GFAP+ astrocytes, which did not appear to have an activated morphology. No brain sections were found that had a condensation of reactive astrocytes suggestive of a gliotic scar. No differences in the pattern of GFAP+ staining was observed in Disomic/mNPC (B) or Trisomic/mNPC (D) mice. Gr, granule cell layer; rad, stratum radiatum; CC, Corpus callosum. Scale Bars in A–D = 500 µm. (TIF) [file pone.0036082.s002.tif]

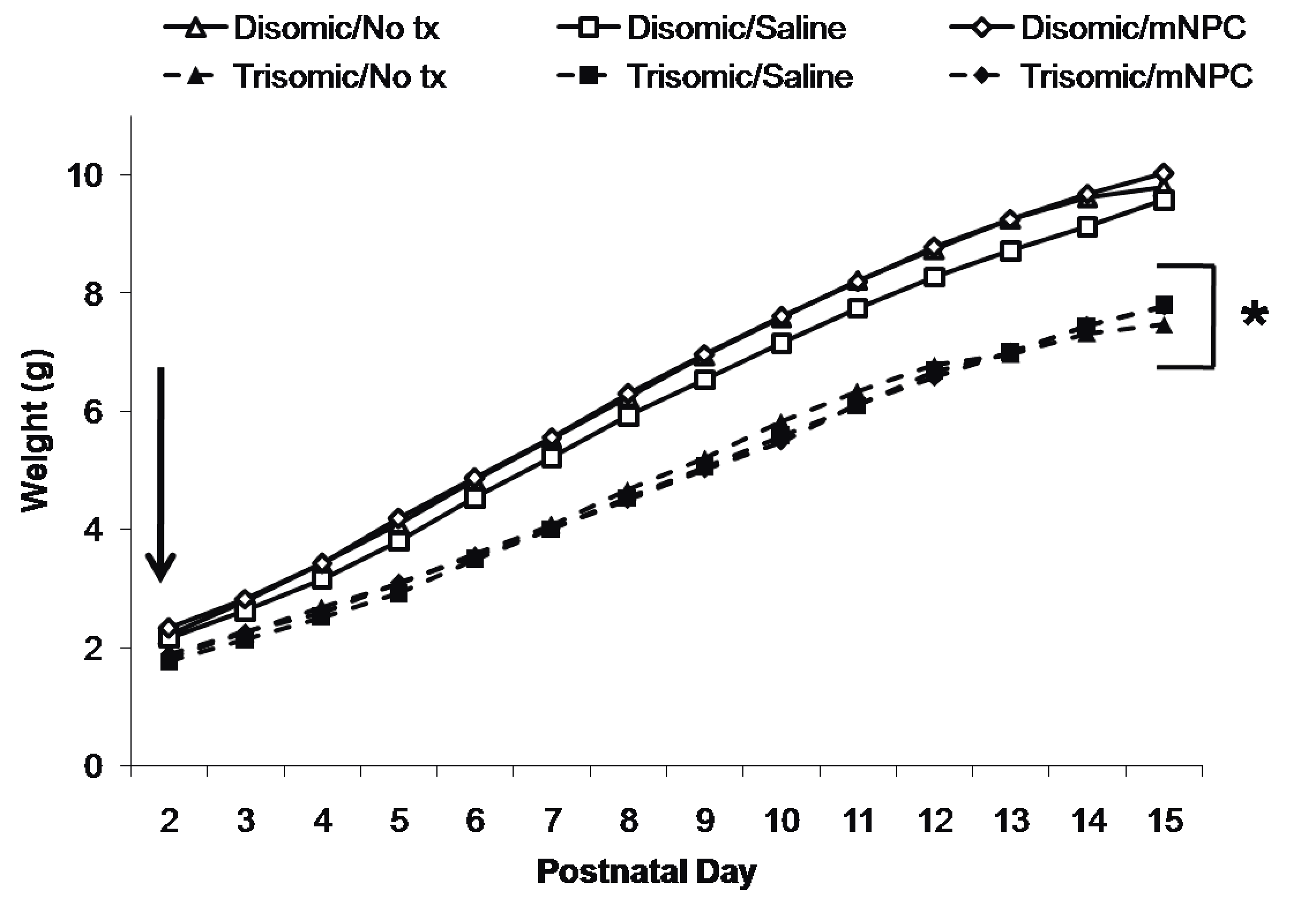

Supplement: Figure S3 — Treatment did not alter weight gain in pups. Typical for trisomic pups, their weights were lower than their disomic littermates (p<0.001). The implantation of saline or mNPC did not alter the weight gain of either group (p>0.05). Mean weight values of each karyotype/treatment group of pups are shown. *Significantly different weight at PND 21 between all trisomic and all disomic groups (main effect of karyotype). ↓ indicates day of treatment. (TIF) [file pone.0036082.s003.tif]

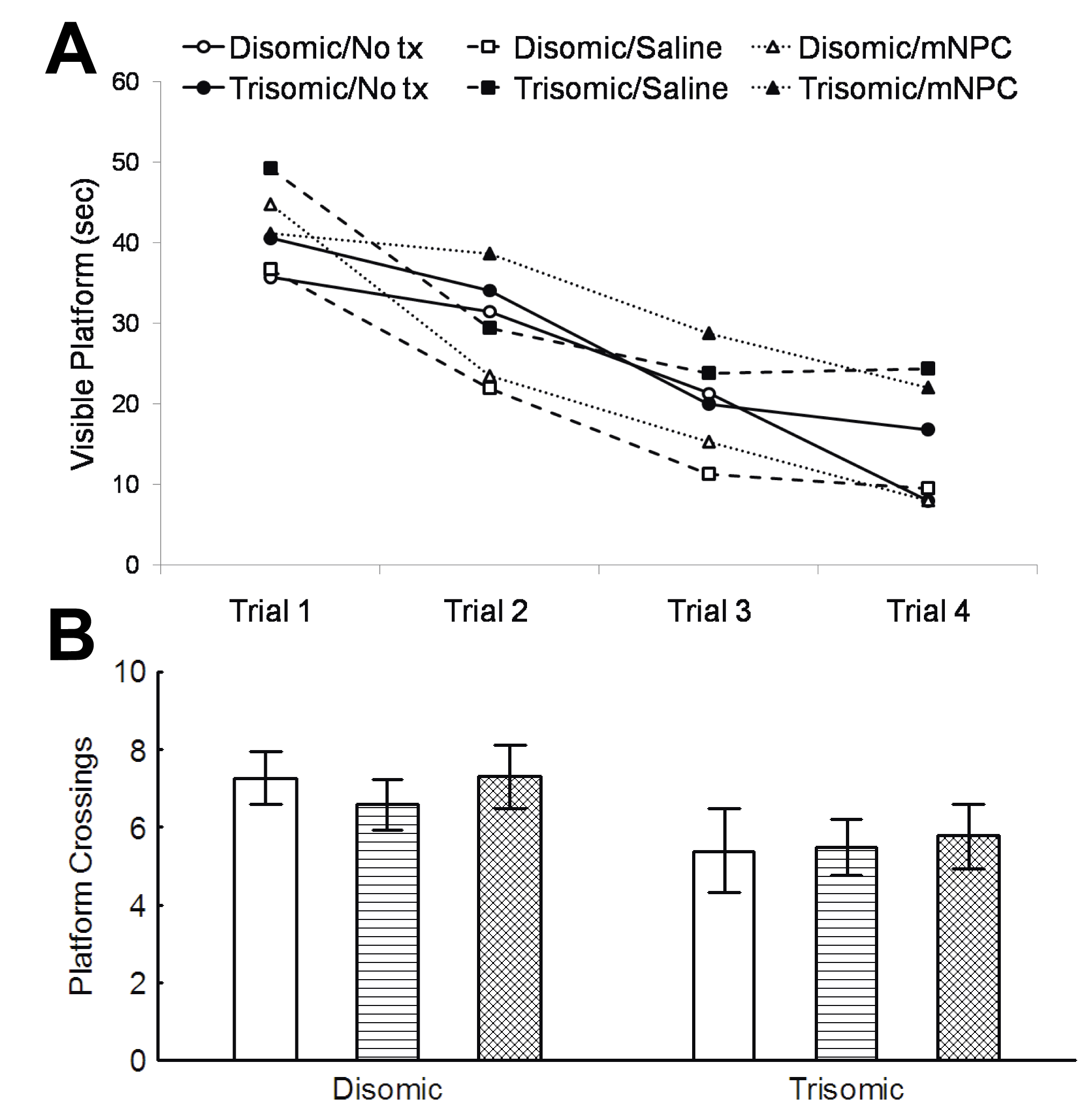

Supplement: Figure S4 — Visible platform training and platform crossings were similar between all groups in the MWM. A. Trisomic mice as a group had longer average latencies to reach the visible platform, however all groups improved their performances over the four trials, as indicated by the parallel lines. B. In the probe trial, all groups had a similar number of platform crossings. No group showed an increased tendency to perseverate in the platform location. Mean ± SEM shown. (TIF) [file pone.0036082.s004.tif]

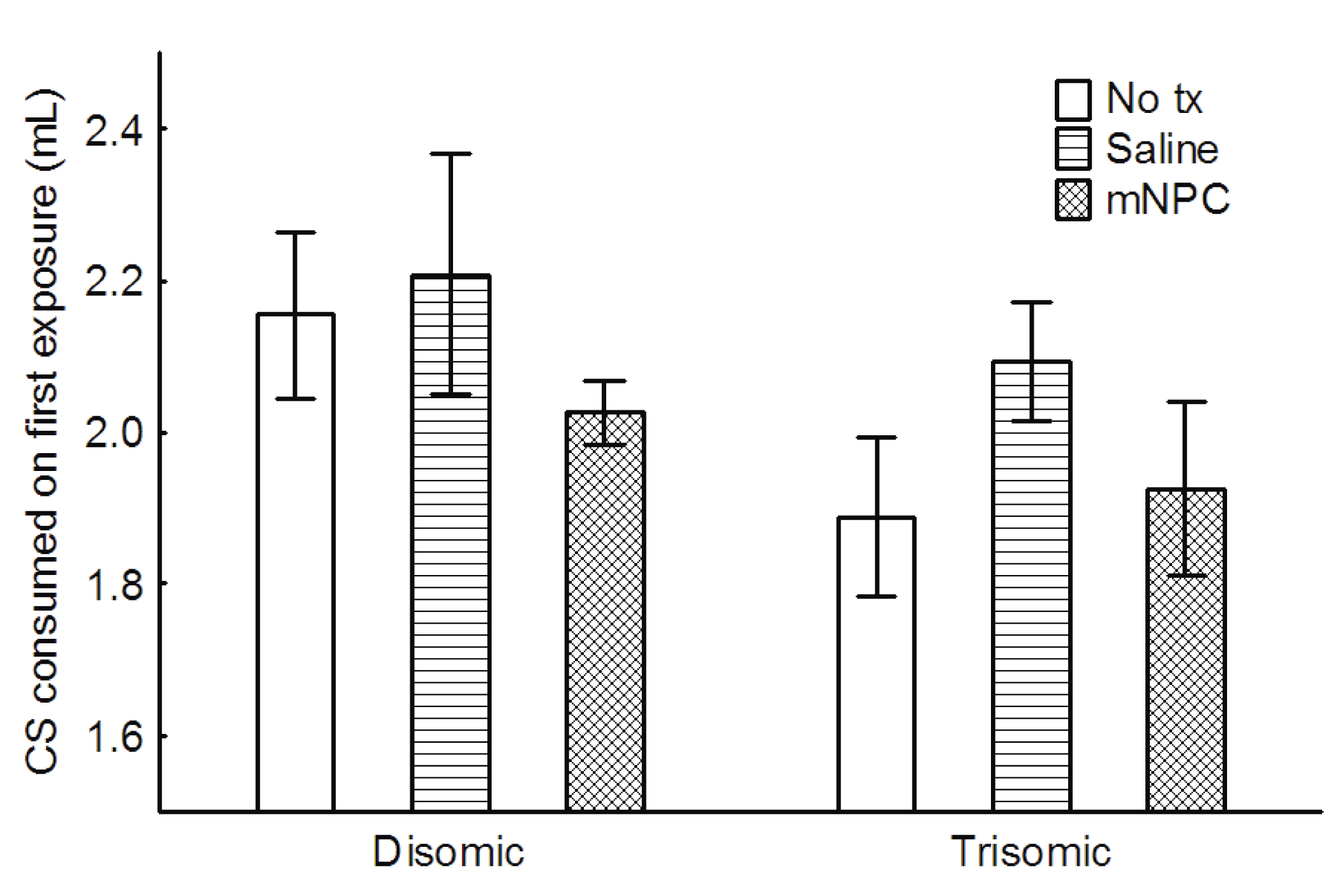

Supplement: Figure S5 — Both disomic and trisomic mice consumed similar volumes of CS on Training Day. There was no significant difference in the amount (mLs) of CS consumed by either karyotype or treatment group on first exposure (p>0.05). Data subsequently was normalized to the average drinking behavior for each animal to account for individual variations. (TIF) [file pone.0036082.s005.tif]

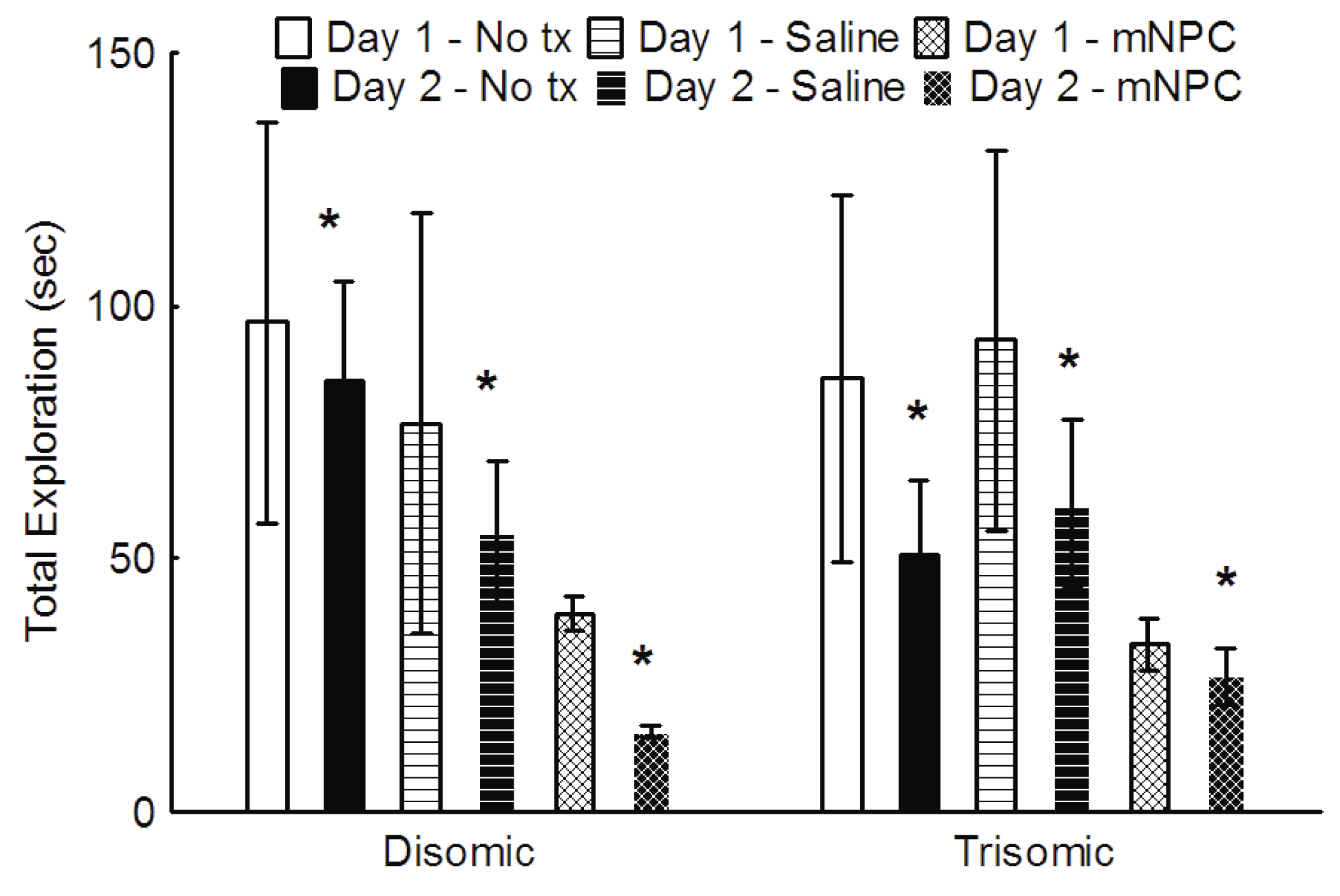

Supplement: Figure S6 — All groups had similar total exploration times in the NOR. There was large intragroup variability on both days of exploration, especially for No treatment and Saline treated groups of both karyotypes. All groups explored significantly less on Day 2. Mean ± SEM shown. (TIF) [file pone.0036082.s006.tif]

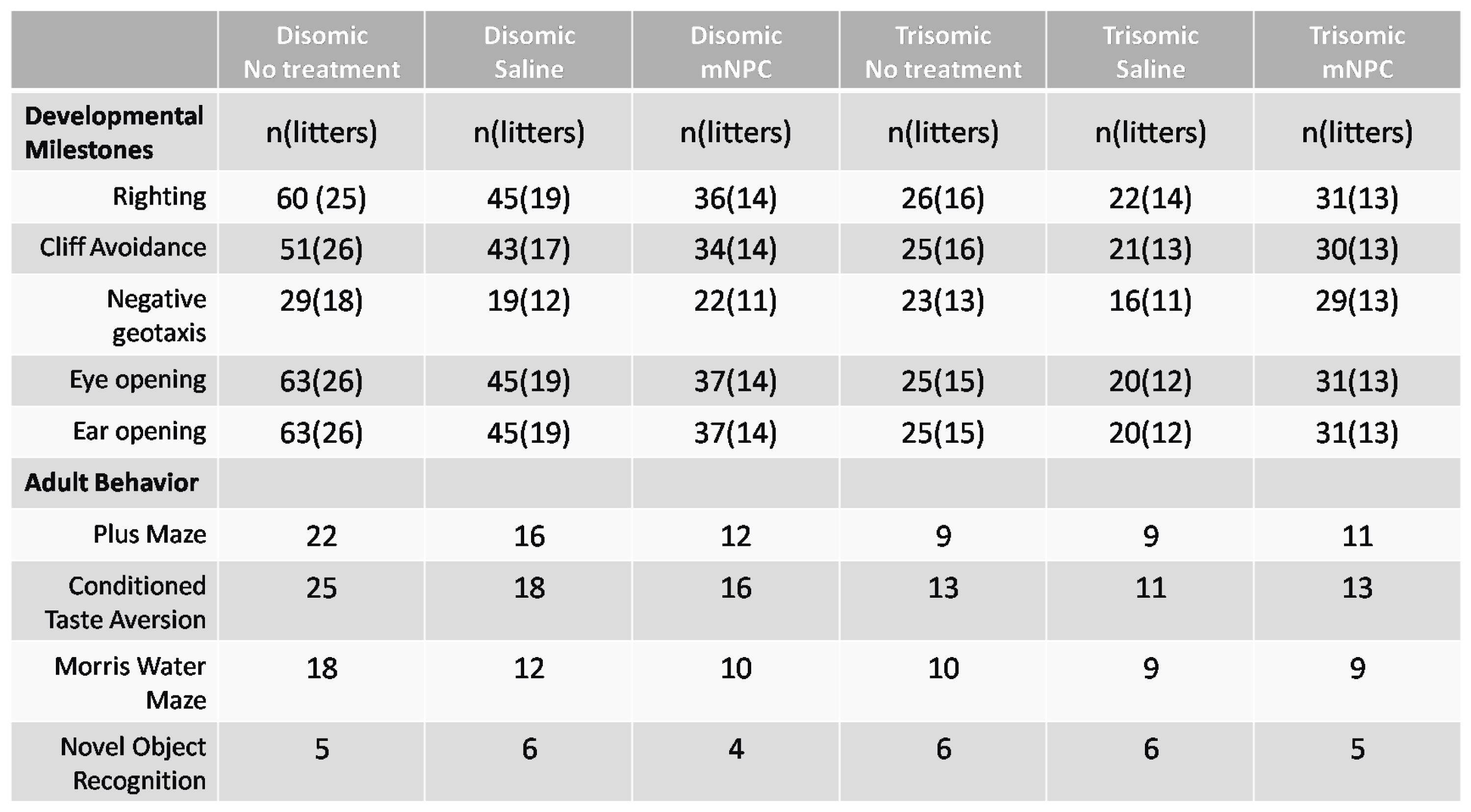

Supplement: Table S1 — Number of animals used in each cognitive test. (TIF) [file pone.0036082.s007.tif]
